# Aneuploidy in targeted endoscopic biopsies outperforms other tissue biomarkers in the prediction of histologic progression of Barrett's oesophagus: A multi-centre prospective cohort study

**DOI:** 10.1016/j.ebiom.2020.102765

**Published:** 2020-05-24

**Authors:** Andreas V. Hadjinicolaou, Sanne N. van Munster, Achilleas Achilleos, Jose Santiago Garcia, Sarah Killcoyne, Krish Ragunath, Jacques J.G.H.M. Bergman, Rebecca C. Fitzgerald, Massimiliano di Pietro

**Affiliations:** a MRC Cancer Unit, University of Cambridge, Hutchison/MRC Research Centre, Box 197, Cambridge Biomedical Campus, Cambridge CB2 0XZ, United Kingdom; b Department of Gastroenterology, Amsterdam University Medical Centre, Meibergdreef 9, 1105 AZ, Amsterdam 22660, the Netherlands; c Nottingham Digestive Diseases Biomedical Research Centre, Queens Medical Centre Campus, E Floor, West Block, Derby Road, Nottingham NG7 2UH, United Kingdom; d European Molecular Biology Laboratory, European Bioinformatics Institute (EMBL-EBI), Hinxton CB10 1SD, United Kingdom

**Keywords:** Barrett's oesophagus, Histologic progression, Dysplasia, Oesophageal adenocarcinoma, Biomarkers

## Abstract

**Background:**

The cancer risk in Barrett's oesophagus (BO) is difficult to estimate. Histologic dysplasia has strong predictive power, but can be missed by random biopsies. Other clinical parameters have limited utility for risk stratification. We aimed to assess whether a molecular biomarker panel on targeted biopsies can predict neoplastic progression of BO.

**Methods:**

203 patients with BO were tested at index endoscopy for 9 biomarkers (p53 and cyclin A expression; aneuploidy and tetraploidy; *CDKN2A* (*p16*), *RUNX3* and *HPP1* hypermethylation; 9p and 17p loss of heterozygosity) on autofluorescence-targeted biopsies and followed-up prospectively. Data comparing progressors to non-progressors were evaluated by univariate and multivariate analyses using survival curves, Cox-proportional hazards and logistic regression models.

**Findings:**

127 patients without high-grade dysplasia (HGD) or oesophageal adenocarcinoma (OAC) at index endoscopy were included, of which 42 had evidence of any histologic progression over time. Aneuploidy was the only predictor of progression from non-dysplastic BO (NDBO) to any grade of neoplasia (*p* = 0.013) and HGD/OAC (*p* = 0.002). Aberrant p53 expression correlated with risk of short-term progression within 12 months, with an odds ratio of 6.0 (95% CI: 3.1–11.2). A panel comprising aneuploidy and p53 had an area under the receiving operator characteristics curve of 0.68 (95% CI: 0.59–0.77) for prediction of any progression.

**Interpretation:**

Aneuploidy is the only biomarker that predicts neoplastic progression of NDBO. Aberrant p53 expression suggests prevalent dysplasia, which might have been missed by random biopsies, and warrants early follow up.

**Research in context**

**Evidence before this study**

Barrett's oesophagus (BO) is a pre-cancerous lesion to oesophageal adenocarcinoma and affects 1.5–2.0% of the Western population. Endoscopic surveillance of BO is recommended with the aim to detect dysplasia and early cancer, which can be treated with minimally endoscopic therapies. However, the risk of progression to cancer in BO is low, hence many patients have unnecessary surveillance procedures. On the other hand, dysplasia is often invisible at endoscopy, therefore patients at higher risk of progression might be under-diagnosed and present later with invasive cancer. Consequently, better tests are required to improve diagnosis and risk stratification. Several retrospective studies assessed the utility of molecular biomarkers, individually or as panels, to improve risk stratification, however there is lack of well-designed prospective studies to inform clinical practice. In a previous cross-sectional study, we tested a large panel of 9 molecular biomarkers on biopsies targeted by autofluorescence imaging and found that a 3-biomarker panel, comprising p53, DNA aneuploidy and cyclin A, has high diagnostic accuracy for prevalent high-grade dysplasia and early cancer in BO. In the present study we evaluated the predictive power of the extended panel of biomarkers in the same patient cohort, which was followed up for a median of 4.6 years.

**Added value of this study**

This is a prospective multicenter study on a large patient cohort with long follow up, precise clinico-pathological annotation and comprehensive molecular biomarker analyses. Our data show that DNA aneuploidy is the only biomarker that can predict long-term neoplastic progression in BO. Furthermore, we show that aberrant p53 correlates with short-term neoplastic progression, suggesting a high risk of histologically missed dysplasia at the time of a negative endoscopy. The combination of aneuploidy and p53 as a molecular panel outperforms current clinical models and could be used in clinical practice to risk stratify patients with BO.

**Implications of all the available evidence**

Our findings have significant clinical implications, in that they indicate that aneuploidy and p53 can be used to inform patient management. Positive biomarkers identify patients with BO at high risk of neoplastic progression. These should be closely followed up with rigorous surveillance, even in absence of histologic dysplasia, and potentially be considered for early endoscopic ablation in the appropriate clinical setting.

Alt-text: Unlabelled box

## 1 Introduction

Barrett's oesophagus (BO) is a precancerous lesion to oesophageal adenocarcinoma (OAC) that affects approximately 1.5–2.0% of the Western population [1], [2], [3]. The incidence of OAC has been increasing in Western Europe, North America and Australia in the last few decades [4]. Given the dismal 5-year survival of OAC (15%) [5], early diagnosis is paramount to improve survival, hence endoscopic surveillance of BO is generally recommended to allow detection of dysplasia [6], [7], [8]. The annual cancer progression rate of non-dysplastic BO (NDBO) is estimated to be around 0.3%/year [9,10], however it increases dramatically in the presence of dysplasia [10], [11], [12], [13], [14]. Therefore, current guidelines recommend endoscopic ablation of BO with dysplasia confirmed by two independent pathologists [7,8,15].

However, current management practice still suffers from several limitations. The accuracy of endoscopic surveillance is affected by the inconspicuous nature of dysplasia and the sampling error arising from random biopsies, which are invasive and time-consuming. Furthermore, the diagnosis and grading of dysplasia is very subjective with low level of inter-observer agreement among pathologists [11,16]. Finally, in the absence of dysplasia, risk prediction tools based on clinical parameters such as sex and BO segment length are not sufficiently accurate [6,[17], [18], [19]]. Therefore, there is increasing need to identify and validate biomarkers that can risk stratify BE patients.

Sequencing data show that genomic aberrations found in OAC can occur as early as non-dysplastic stage BO and increase in cancer, which provides support to a risk stratification strategy with molecular biomarkers [20,21]. In previous retrospective case-control studies, tissue biomarkers that showed good level of prediction power include loss of heterozygosity (LOH) at p16 and p53 loci, DNA aneuploidy/tetraploidy, aberrant expression of p53 and cyclin A proteins and some methylation markers [22], [23], [24], [25], [26]. In particular immunohistochemistry for p53 and cyclin A have the advantage of being easily applied to standard clinical specimens. Combining biomarkers into a panel is also a viable strategy to increase the prediction accuracy. In a large population-based case-control study, a panel combining aneuploidy, aspergillus oryzae lectin (AOL) IHC and low-grade dysplasia (LGD) were the most predictive with an area under the receiver operating curve (AUC) of 0.75 for histologic progression [27]. Another retrospective case-control study showed that expert LGD, AOL and p53 formed the best predictive panel with an AUC of 0.73, [28]. However, retrospective studies are subject to patient selection bias, high degrees of missing data and less rigorous sample selection.

A recent prospective study, which assessed chromosomal aberrations by fluorescence in situ hybridization on brush cytology samples, found that a panel of 3 markers (p16, MYC and aneusomy) predicted progression to HGD/OAC with an AUC of 0.76 (95% CI 0.66–0.86) [29]. Although the results from this well designed study are encouraging, the methodology required to assess chromosomal alteration is laborious and difficult to adapt to routine pathology laboratories.

So far the endpoint for these studies has been HGD/OAC and none of biomarkers has been validated for prediction of progression to LGD. LGD is now an endpoint for endoscopic therapy [7,8,15] given its significant risk of progressing to OAC [11,15,30,31] and hence biomarkers are also required to confidently identify patients at risk for any dysplasia.

These biomarker studies have generally been conducted on random biopsies, which may miss areas of inconspicuous dysplasia due to sampling error. Image-enhanced modalities such as autofluorescence imaging (AFI), acetic acid chromoendoscopy or narrow band imaging (NBI) can increase detection of inconspicuous dysplasia. Despite advances in endoscopic imaging, there is lack of evidence that this is feasible and effective in routine practice given the training and operator dependence of these modalities, hence white light high-resolution endoscopy remains the gold standard [13,32,33]. However, if an imaging modality could be used to help target the biopsies, this might reduce the number of samples required without the endoscopist being required to rely on the image for a virtual dysplasia diagnosis. Recently we conducted a multi-centre study in a large cohort of patients with BE and used AFI to obtain a small number of targeted biopsies for evaluation of a large panel of nine different molecular biomarkers with the aim to improve detection of prevalent dysplasia. In the cross-sectional phase of this study a panel of 3 biomarkers (aneuploidy, p53 and cyclin A) diagnosed prevalent HGD/OAC with an AUC of 0.97 (95% CI: 0.95 to 0.99) [34]. Patients with no HGD/OAC at index endoscopy and those who did not receive endoscopic ablation for LGD were offered prospective endoscopic follow up.

In light of these promising data this prospective study aimed to extend the imaging-targeted biomarker approach used for identification of prevalent dysplasia to predict neoplastic progression (incident disease). To do this we tested a biomarker panel in AFI-targeted biopsies in patients with BO and followed them up to evaluate the optimal marker(s) for progression to LGD as well as HGD/OAC.

## 2 Material and methods

### 2.1 Study design

This was a prospective study approved by the Cambridgeshire 2 Research Ethics Committee (09/H0308/118) and the Amsterdam University Medical Centre (AUMC) Medical Ethics Committee (MEC 09/073). This was a National Institute of Health research (NIHR) portfolio study (UKCRN ID 7561). Patients were recruited for an index endoscopy at three tertiary referral centres for BO between April 2009 and April 2014 and were followed up with repeat endoscopies and biopsies in accordance with the local BE surveillance guidelines until February 2019. Written consent was obtained according to the Declaration of Helsinki. Endoscopic and histological findings for each patient were recorded locally in a prospective database. Time of follow-up was defined as the period between index endoscopy and the most recent surveillance endoscopy for non-progressors or, the period between index endoscopy and the procedure that detected early neoplasia, for patients with evidence of histological progression. Histological progression was defined as transition from a NDBO or indefinite for dysplasia (ID) to any dysplasia, or if low-grade dysplasia already present, to a higher grade of dysplasia or cancer. The primary endpoint of this study was progression from NDBO/ID to any grade of dysplasia. The two secondary endpoints were a) progression from NDBO/ID to HGD/OAC, and b) any histologic progression i.e. NDBO/ID to LGD, NDBO/ID to HGD, and LGD to HGD.

**Fig. 1:**
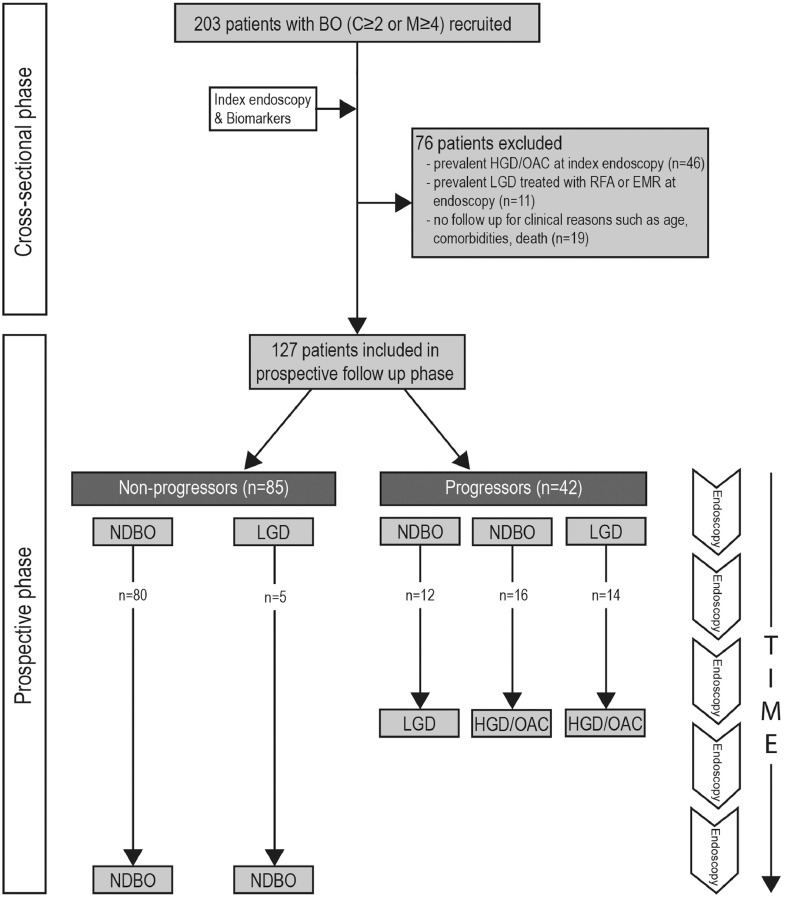
Flow chart schematic for patient eligibility and follow up for included patients in the study depicting progressors and non-progressors. BO, Barrett's oesophagus; RFA, radiofrequency ablation; EMR, endoscopic mucosal resection; ND, non-dysplastic; LGD, low-grade dysplasia; HGD, high-grade dysplasia; OAC, oesophageal adenocarcinoma.

### 2.2 Inclusion and exclusion criteria

Patients older than 18 years were included if they were referred for evaluation of BO with a length of at least *C* ≥ 2 or *C*<2M≥4 according to the Prague classification with or without visible lesions [35]. BO was defined as the presence of metaplastic mucosa on endoscopy with histologic evidence of intestinal metaplasia (presence of goblet cells). Patients were excluded at baseline if they had oesophagitis of grade B or above (according to Los Angeles classification), previous upper gastrointestinal (UGI) surgery (except Nissen fundoplication), UGI tract anatomical anomalies, coagulopathies or high risk conditions requiring continued anticoagulant/antiplatelet medications, active or severe cardiopulmonary or liver disease, dysphagia or special communication needs. Patients with (i) at least one follow-up endoscopy with biopsy results and (ii) no evidence of HGD/OAC at index endoscopy, were included in the follow up phase of the study. Patients that received treatment with RFA or EMR at index endoscopy or at the immediate follow-up endoscopy were excluded from the follow up phase.

### 2.3 Endoscopic procedure, biopsy and histopathology

The index upper gastrointestinal endoscopy was performed as previously described [34]. Briefly, FQ260Z endoscopes (Olympus Inc, Tokyo, Japan) were used by endoscopists with experience in AFI imaging [13]. Up to four AFI-positive areas, as well as one AFI-negative control area were selected for targeted biopsies, followed by Seattle protocol biopsies. Histology on index endoscopy was based on the combination of AFI-targeted biopsies and quadrantic random biopsies. Histological assessment was performed according to the Vienna classification by an expert GI pathologist and, in cases with any grade dysplasia, further reviewed by a second study pathologist to reach consensus [36]. At follow-up endoscopies, biopsies were taken according to the Seattle protocol. Molecular biomarker analysis was carried out on AFI-targeted biopsies only.

### 2.4 Molecular biomarker analysis

A panel of nine molecular biomarkers was evaluated as previously described [34]. A mean of 2.8 biopsies per patient were used for molecular analysis. Briefly, p53 and cyclin A were analysed by immunohistochemistry (IHC); aneuploidy and G2/tetraploidy, were analysed by flow cytometry; p16, RUNX3 and HPP1 hypermethylation was analysed by quantitative methylation-specific PCR (Methylight); and LOH at 9p and 17p loci was analysed by the use of microsatellite markers. Snap frozen biopsies in DMSO were used for aneuploidy, G2 tetraploidy, LOH markers and methylation assays. Since not all biomarkers could be tested in each biopsy due to limited material, biopsies from individual patients were randomly allocated to different biomarkers. However, a proportion of patients recruited between March 2012 and April 2014 (*n* = 46) were tested for aneuploidy, p53 and cyclin A only, as this was a validation cohort from the cross-sectional study. The molecular analyses were performed at the MRC Cancer Unit (Cambridge, UK). Briefly, p53 immunohistochemistry (IHC) staining was carried out using the BOND™ System (Leica Microsystems, Ltd, Milton Keynes, UK) using anti-p53 antibody (p53 clone DO7, Dakocytomation, 1:50 dilution). p53 was scored positive in the presence of one of two aberrant patterns, i.e. strong staining or complete absence of staining (Supplementary Fig. 1). Anomalies in DNA content were analysed in nuclei isolated from snap frozen biopsies preserved in DMSO using either MoFlow (Beckman Coulter, Miami, FL, USA) or BD Influx™ (Becton, Dickenson biosciences, New Jersey, USA). ModFit LT software (Verity Software House, Topsham, ME, USA) was used to generate cell cycle histograms. The presence of separate populations of nuclei deviating from the standard G1 peak profile was interpreted as aneuploidy. The details of the molecular analyses of other biomarkers have been previously reported [34].

### 2.5 Statistical analysis

Univariate survival analysis was performed using Kaplan–Meier (K–M) plots. We examined all biomarkers and their interaction terms. Evaluations in a multivariate context were carried out using a Cox proportional hazards model (R-package: survival) or logistic regression (R-package: stats) for binary outcomes and using multinomial logistic regression for categorically distributed dependent variables. Similar findings were observed using logistic and Cox proportional hazards regression analysis, hence in our Results section, we use odds ratios obtained from the former model. As part of our stepwise regression analysis, we also used a backward variable selection method with a significance level threshold set to 0.05 for variables to enter the model. In brief, the process starts with inclusion of all candidate variables in the model followed by the removal of the covariate with the least significant p-value at each subsequent step. This is repeated until no non-significant variables remain. The resulting model should only contain variables that are statistically significant, if any. Receiver operating characteristic curve analysis was performed using the R-package (pROC). Missing data was imputed using nonparametric imputation (R-package: *missForest*). Bonferroni correction was applied on p-values to adjust for multiple comparisons (Table 1). Confidence intervals for proportions were calculated using the Clopper and Pearson method (R-package: stats). P-values less than 0.05 were considered statistically significant.

**Table 1:** Baseline characteristics of patient cohort comparing progressors (any progression) vs non-progressors.

Variable	Total patient population (*n* = 127)	Progressors (*n* = 42)	Non-progressors (*n* = 85)	Progressors vs non-progressors comparison
M:F (ratio)	107:20 (5.4:1)	36:6 (6:1)	71:14 (5.1:1)	*p* = 0.98 (Fisher Exact test)
Median age in yrs (Q1–Q3)	65.6 (59.2–72.9)	64.9 (58.5–68.9)	66.0 (60.3–73.0)	*p* = 0.91 (*t*-test)
Median BO length in cm (Q1–Q3)	6 [5–9]	6 [5–9]	7 [5–9]	*p* = 0.94 (*t*-test)
Median follow-up in yrs (Q1–Q3)	4.6 (2.0–6.3)	1.2 (0.6–3.3)	5.4 (4.0–6.5)	*p* = 0.000003 (*t*-test)
Median number of AFI+ areas (Q1–Q3)	1 [1–2]	1 [1–2]	1 [1–2]	*p* = 0.97 (*t*-test)
Baseline histology NDBO:ID:LGD	98:10:19	24:4:14	74:6:5	*P* = 0.00015 (Fisher Exact test)

M, male; F, female; BO, Barrett's oesophagus; AFI, autofluorescence imaging; NDBO, non-dysplastic Barrett's oesophagus; ID, indefinite for dysplasia; LGD, low-grade dysplasia.

## 3 Results

A total of 203 patients with BO received an index endoscopy and molecular biomarker analysis. Of these, 76 (37.4%) patients were excluded from the final analysis due to either the presence of HGD or OAC at baseline (*n* = 46), or treatment received for prevalent LGD in the form of RFA or EMR (*n* = 11) or to lack of follow-up endoscopy due to old age, comorbidities, relocation to a different city or death (*n* = 19) (Fig. 1), leaving 127 (62.6%) patients for inclusion in the final analysis. The final study cohort had a median age of 65.6 years (IQR, 13.7 yrs), with a median follow-up of 4.6 yrs (IQR, 4.3 yrs) per patient. The majority (83.5%) of the patients were male. The median BO length was 7.0 cm (IQR, 4.0 cm). A total of 182 AFI+ areas with corresponding molecular data were included in the analysis, of which only 28 (15.4%) related to subtle visible lesions on white light endoscopy.

For the duration of this study, there were 42 (33.1%) patients that were diagnosed with histologic progression during follow up. The median follow-up from index endoscopy to progression was 1.2 yrs (IQR, 2.7 yrs). The comparison between baseline characteristics of progressors (any progression) and non-progressors is shown in Table 1. The two groups were overall well matched in terms of demographics. As expected there was a higher proportion of patients with LGD at baseline among those that had any histological progression (*p* = 0.00015; Fisher Exact test). Post-hoc pairwise analysis with adjustment for multiple comparisons confirmed that only the proportion of baseline LGD was significantly different between progressors and non-progressors. This suggests that the histological diagnosis of ID had no effect on the association between biomarker status and histologic progression. Amongst progressors, there were 12 (28.6%) that progressed from NDBO/ID to LGD, 16 (38.1%) that progressed from NDBO/ID to HGD/OAC and 14 (33.3%) that progressed from LGD to HGD/OAC. The rate of any progression was 0.08 (95% CI: 0.06–0.11) per person-year. The rate of progression was 0.02 (95% CI: 0.01–0.04) per person-year for NDBO/ID to LGD, 0.03 (95% CI: 0.02–0.05) per person-year for NDBO/ID to HGD/OAC and 0.43 (95% CI: 0.26–0.62) per person-year for LGD to HGD. The results of the individual biomarkers in the progressors and non-progressors are presented in Supplementary Table 1.

**Fig. 2:**
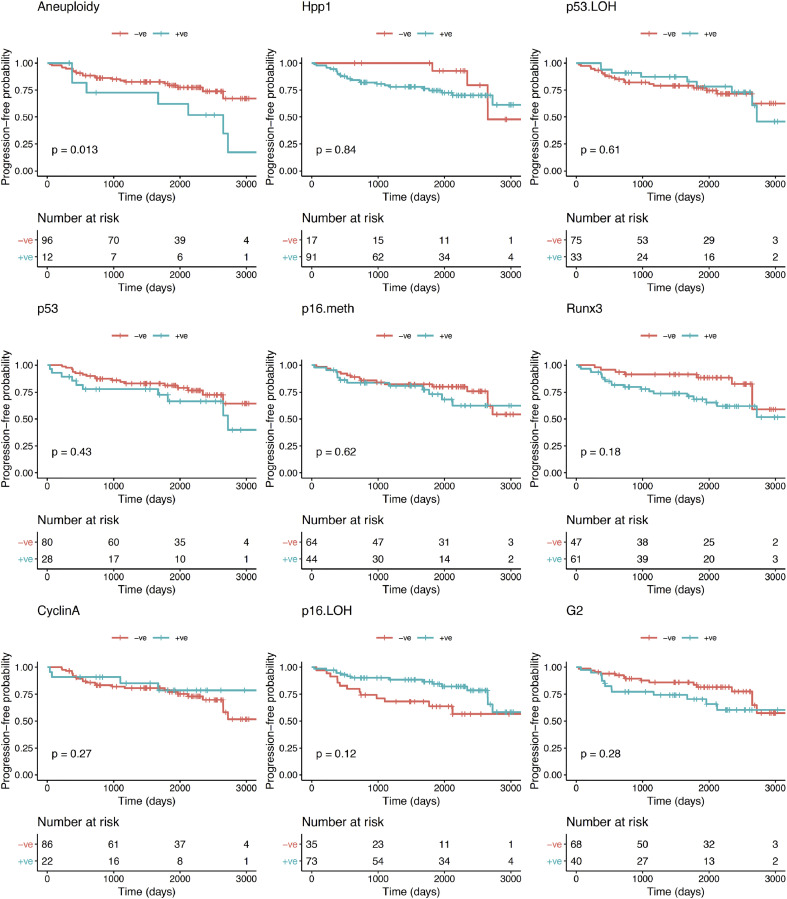
Kaplan–Meier curves for each biomarker for progression-free survival probability of histological progression from NDBO/ID to LGD/HGD/OAC.

**Fig. 3:**
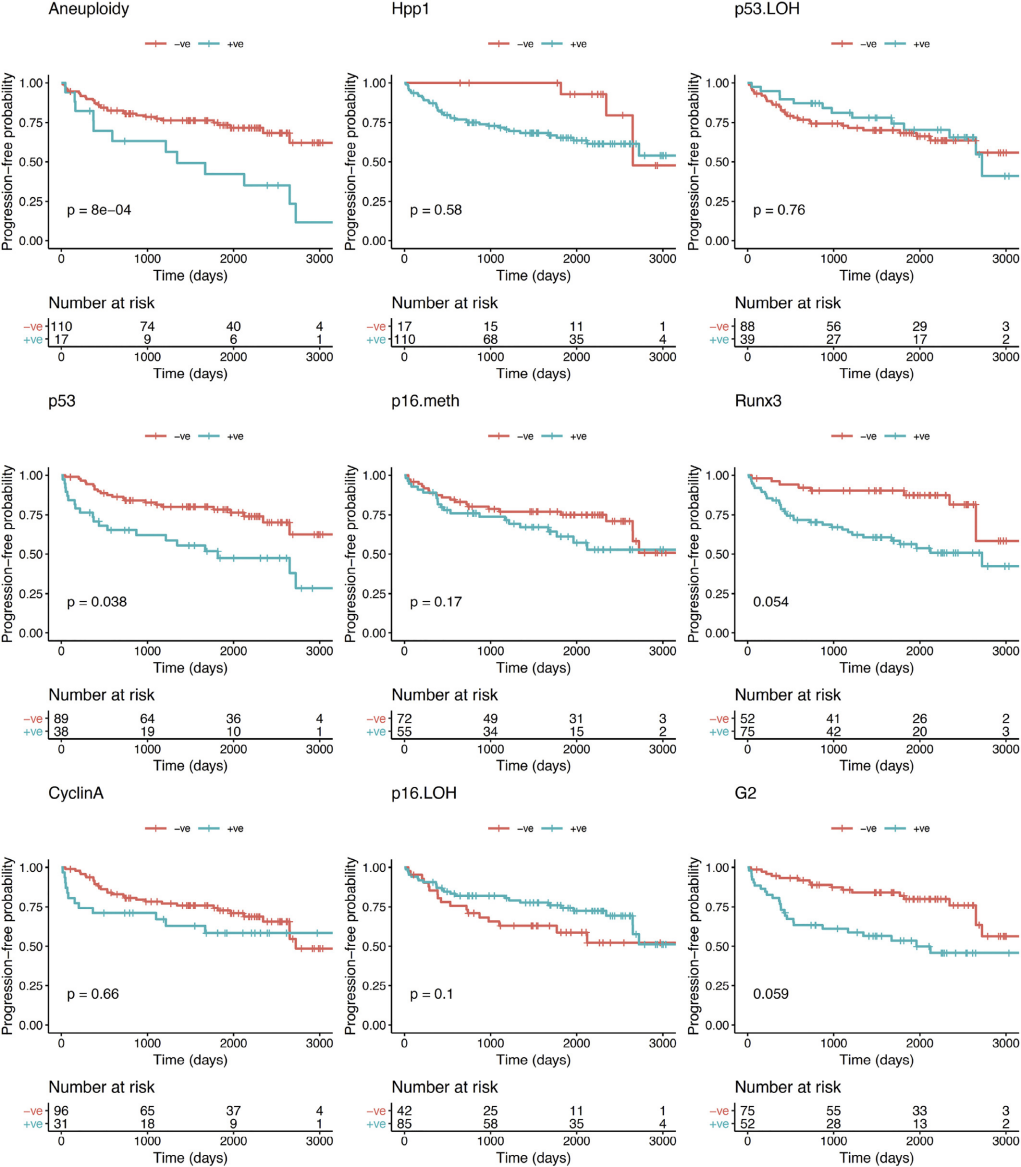
Kaplan–Meier curves for each biomarker for progression-free survival probability of any histological progression (NDBO/ID to LGD/HGD/OAC and LGD to HGD/OAC).

**Fig. 4:**
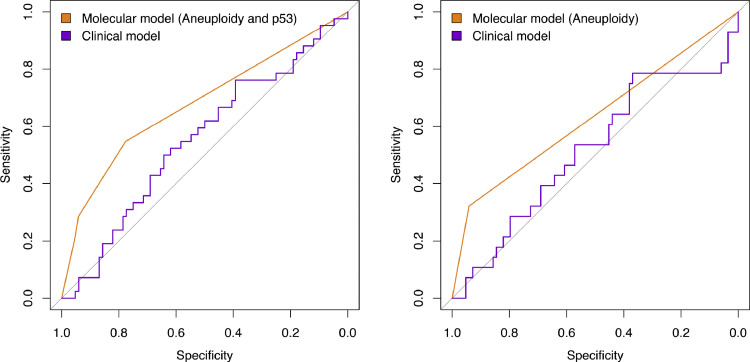
Receiver operating characteristic curves for a clinical model (age and Barrett's length) vs molecular model. Left panel) Analysis on all progressors: molecular model includes aneuploidy and p53 with a cutoff of at least one positive biomarker; Right panel) Analysis excluding progressors within 12 months of index endoscopy: molecular model includes only aneuploidy.

With regards to the primary endpoint of the study (progression to any grade of dysplasia), the univariate analysis showed that, of the biomarkers and clinical variables evaluated at initial OGD, aneuploidy was the only variable that significantly correlated with progression from NDBO/ID to any grade of neoplasia (*p* = 0.013; Log-rank) (Fig. 2). With reference to the secondary endpoints, aneuploidy had a significant effect on progression probability (1 - probability of progression-free survival) from NDBO/ID to HGD/OAC (*p* = 0.002; Log-rank). However, analysis of the data related to the other secondary endpoint (any histological progression) aneuploidy (*p* = 0.0008; Log-rank) and p53 (*p* = 0.038; Log-rank) were significant predictors of progression (Fig. 3). To further evaluate the progressors’ cohort, we analysed clinical and molecular variables, looking at any progression, using a Cox proportional hazards model and logistic regression. Backward model selection confirmed that p53 and aneuploidy were the only significant predictors of any progression with cyclin A behaving as a negative confounder of p53 (Supplementary Table 2). Furthermore, receiver operating characteristic curves (ROC) showed that a clinical model using patient age and BO length (AUC=0.55; CI: 0.45–0.66) was outperformed in the prediction of any histologic progression by a molecular biomarker model comprising of aneuploidy and p53 with a cut-off of one positive biomarker out of two (AUC=0.68; CI: 0.59–0.77, Fig. 4, left panel). Combining clinical and molecular parameters in a single model did not improve the sensitivity or specificity of predicting histologic progression. We also looked at whether the number of biopsies or endoscopic areas affected the rate of biomarker positivity. Comparison of patients with ≤2 AFI+ areas with those with ≥3 AFI+ areas did not reveal significant differences for biomarker positivity rate for neither p53 (28% vs 33%; *p* = 0.6111) nor aneuploidy (11% vs 14%; *p* = 0.7136).

**Fig. 5:**
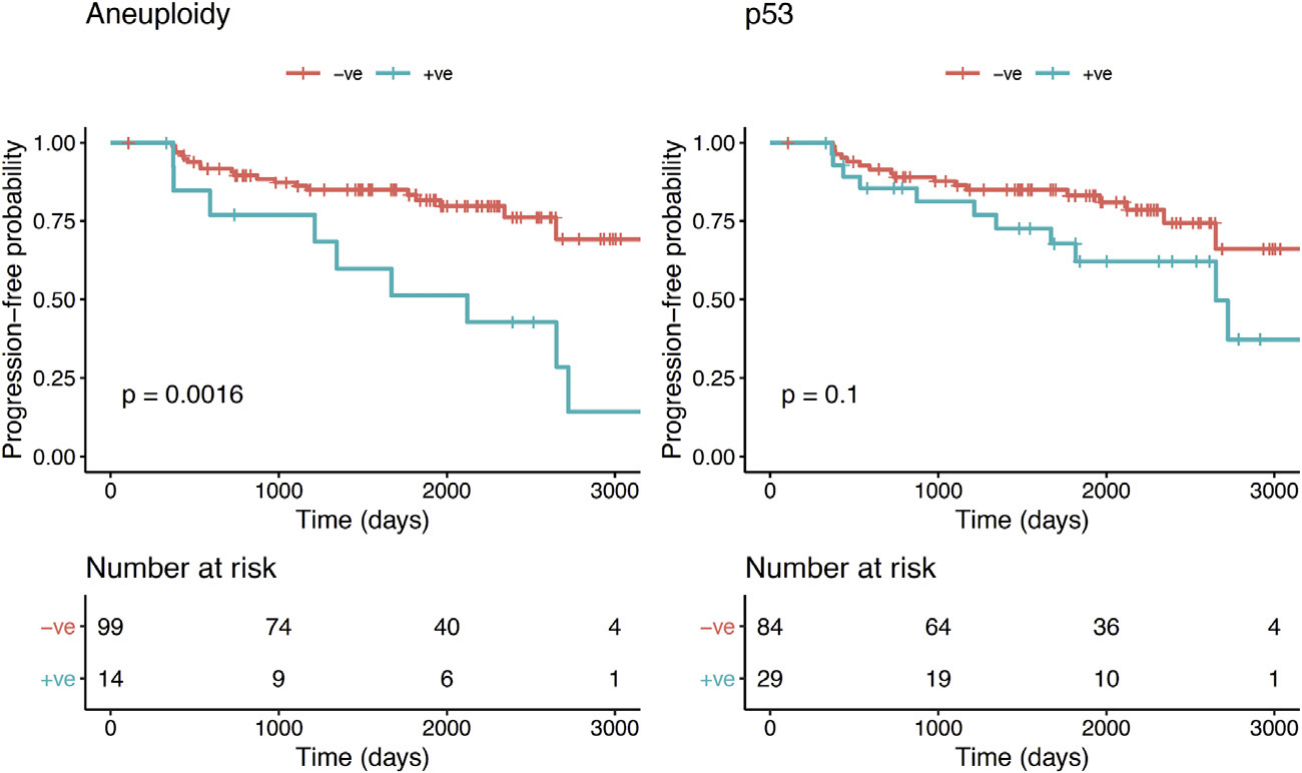
Kaplan-Meier curves for aneuploidy (left) and p53 (right) for progression-free survival probability of any histological progression excluding early progressors (progression within 12 months of index endoscopy).

A proportion of patients displayed histological progression within 12 months from the index endoscopy, suggesting that they may have had prevalent dysplasia at time 0 (*n* = 14). Therefore, in a sensitivity analysis, we excluded them from the progressors’ cohort. Kaplan–Meier analysis revealed that aneuploidy remained significant in predicting any progression (*p* = 0.0016; Log-rank), but p53 lost its significance (*p* = 0.1; Log-rank) (Fig. 5). We confirmed these findings using a multinomial logistic regression model to adjust for other covariates (Table 2). ROC analysis showed that a model with aneuploidy as the only predictor of dysplastic progression outperformed the clinical model (AUC=0.63; CI: 0.54–0.72, Fig. 4, right panel). The presence of positive aneuploidy at index endoscopy led to a 6.6-fold higher risk of dysplastic progression over no progression (95% CI: 1.8–24.8, *p* = 0.005; Z-test; Table 2). Since in our data, p53 appeared to correlate more with short-term progression, we looked at the risk of missed dysplasia in the presence of positive p53 immunostaining. Patients with aberrant p53 expression at index endoscopy had an odds ratio of 6.0 (95% CI: 3.1–11.2, *p* = 0.007; *Z*-test) of missed dysplasia on endoscopic biopsies (Table 2).

**Table 2:** Estimated effects (coefficients) of p53 and aneuploidy variables in a multinomial logistic regression model.

	Natural log Odds Ratio value (*p*-value)		
	Intercept	p53 +ve	Aneuploidy +ve
**Progression**	−1.49 (<0.0001)	0.27 (0.63)	1.89 (0.0051)
**Missed diagnosis**	−2.62 (<0.0001)	1.77 (0.0066)	0.63 (0.46)

Missed diagnosis refers to prevalent dysplasia detected within 12 months from index endoscopy. The intercept values represent the natural log odds ratio values for the two component models (progression vs no progression and missed diagnosis vs no progression, respectively) when both aneuploidy and p53 values are set to zero, i.e. the natural log odds ratios when both aneuploidy and p53 are negative.

## 4 Discussion

Over the last two decades, the increasing knowledge in the molecular events accompanying the development of OAC in patients with BO has led to a body on research aimed to identify biomarkers that can predict clinical behaviour of the pre-malignant disease. Such tests would be extremely useful to guide the decision-making process, including intervals of endoscopic surveillance and timing of endoscopic therapy. The vast majority of research in this field has been conducted on retrospective cohorts of patients, which limited the clinical significance, with very scant availability of prospective studies. In this prospective study, we evaluated the predictive power of nine biomarkers on tissue biopsies guided by advanced imaging, which were previously assessed in a cross-sectional study as markers of dysplasia.

In our study, we found that clinical variables, such as BE length and patient age, were poor predictors of histological progression. This result might have been influenced by the fact that we included patients with long BO segments, therefore selecting for patients with a higher baseline risk.

Of the nine molecular biomarkers investigated, we found that aneuploidy and aberrant p53 expression by IHC were the only ones that showed correlation with histological progression. The two biomarker-based models for prediction of progression outperformed the clinical model based on age and BO length. However, when we excluded patients with progression within 12 months of follow up (prevalent dysplasia), only aneuploidy retained statistical significance. Patients with aneuploidy had 6.6-fold increased risk of neoplastic progression, however the sensitivity of the test to predict progression was low (32%, 95% CI: 16–52%; Fig. 4). This indicates that, while a positive test would warrant an early ablation strategy, a negative test does not allow prolongation of surveillance intervals compared to the current guidelines recommendation. Our data are in agreement with previous cohort studies were aneuploidy was a strong predictor of progression to OAC [22,37]. On the other hand, p53 correlated strongly with short-term progression and the presence of prevalent dysplasia. Patients that had aberrant p53 were 6-fold more likely to progress in the short term or harbour dysplasia, which was missed at index endoscopy. As such, these findings support and highlight previous reports from our group that suggest that p53 is a strong biomarker for prevalent dysplasia in cross-sectional studies [34].

One of the main issues of a biomarker-based clinical strategy is the practical feasibility of the molecular test in routine clinical practice. The advantage of p53 is that immunohistochemistry on formalin-fixed paraffin-embedded (FFPE) biospies is practical and easy to carry out routinely and several centres already use it as part of routine diagnostic process [38]. With regards to aneuploidy, even though in our cross-sectional study we used flow cytometry on fresh biopsies, image cytometry on FFPE material has been validated as an alternative technique and is compatible with routine biopsies [39].

Another important issue with a biomarker-based strategy is how to sample large areas of BO for molecular analyses. It would not be practical or cost effective to process the entire Seattle protocol biopsy set for biomarkers assays. In this study, we used AFI to flag areas for molecular analysis. In our previous cross-sectional study we demonstrated that AFI increased the yield of molecular biomarkers independently of the presence of histological dysplasia [34]. This is promising as it suggests that image-enhanced endoscopy with targeted biopsies can select areas with high molecular instability, where biomarkers are likely to be enriched. This is particularly important as BO-related neoplasia is known to be molecularly heterogenous and therefore we expect differences in the biomarker status among separate biopsies within the same patient [40]. Given that the availability of AFI is not widespread, alternative flagging techniques such as NBI and acetic acid chromoendoscopy should be tested in the future. Alternatively, strategies for wide field sampling could be used. In a previous prospective study, Timmer et al. used endoscopic brushings to cover larger mucosal surface and increase the biomarker yield [29]. The novel wide-area transepithelial sampling device (WATS 3D) is a promising tool, which could be combined in the future with molecular biomarkers [41,42].

This study has some limitations. Firstly, we investigated a tertiary care selected cohort with long segment BO, which might not reflect the general population of patients on endoscopic surveillance. As a result of this, we observed a high progression rate with a number of patients developing dysplasia within 12 months of follow up and a higher rate of patients with baseline LGD among the patients who progressed. However, our primary endpoint referred to neoplastic progression among patients with baseline NDBO only. In addition, we performed a sub-analysis excluding patients with early progression within 12 months to eliminate this bias. Second, the exclusion of early progressors in the sensitivity analysis led to a relatively small number of true progressors, which might in turn have affected the statistical power for finding weaker associations. Finally, some of the biomarkers data was missing due to limited amounts of biopsy material at index endoscopy precluding assessment of the full biomarker set in all patients and a sub-group of patients having only 3 biomarkers evaluated. To overcome this issue, we applied a rigorous imputation methodology to account for the missing data.

In conclusion, this prospective study shows that a biomarker-based approach outperforms the clinical model based on age and BO length for prediction of histological progression. Aneuploidy is the only biomarker with significant predicting power for progression from NDBO/ID to HGD or cancer, while aberrant p53 correlates with prevalent dysplasia, even if missed by histological sampling. The combination of aneuploidy and p53 can be used as a panel to identify, even in the absence of histologic dysplasia, patients at high risk of neoplastic progression, who should undergo closer endoscopic follow up or, in selected cases, be considered for early endoscopic intervention.

## Declaration of Competing Interest

KR reports grants, personal fees and non-financial support from 10.13039/100009734Olympus, outside the submitted work. JB reports grants from Olympus Endoscopy, outside the submitted work. Other authors have nothing to disclose.

## Appendix Supplementary materials

Image, application 1
