# Supplementary material for: Aneuploidy in targeted endoscopic biopsies outperforms other tissue biomarkers in the prediction of histologic progression of Barrett's oesophagus: A multi-centre prospective cohort study
Source: eBioMedicine. 2020 May 24;56:102765. doi: 10.1016/j.ebiom.2020.102765 (PMC7251385; doi:10.1016/j.ebiom.2020.102765)
Supplement: Supplementary file 1 [file mmc1.docx]

**Supplementary Table 1.** Biomarker status according to baseline histology group.

|  | **p53 +ve (%)** | **Aneuploidy +ve (%)** | **cyclinA +ve (%)** | **p16.meth +ve (%)** | **G2 +ve (%)** | **hpp1.meth +ve (%)** | **runx3.meth +ve (%)** | **p53.loh +ve (%)** | **p16.loh +ve (%)** |
| --- | --- | --- | --- | --- | --- | --- | --- | --- | --- |
| **NDBO (non-progressors) n=74** | 14 (18.9) | 3 (4.1) | 15 (20.3) | 27 (36.5) | 25 (33.8) | 61 (82.4) | 35 (47.3) | 23 (31.1) | 52 (70.3) |
| **NDBO (progressors) n=24** | 8 (33.3) | 7 (29.2) | 4 (16.7) | 10 (41.7) | 11 (45.8) | 20 (83.3) | 17 (70.8) | 8 (33.3) | 13 (54.2) |
| **ID (non-progressors) n=6** | 4 (66.7) | 2 (33.3) | 3 (50.0) | 5 (83.3) | 2 (33.3) | 6 (100.0) | 6 (100.0) | 1 (16.7) | 6 (100.0) |
| **ID (progressors) n=4** | 2 (50.0) | 0 (0.0) | 0 (0.0) | 2 (50.0) | 2 (50.0) | 4 (100.0) | 3 (75.0) | 1 (25.0) | 2 (50.0) |
| **LGD (non-progressors) n=5** | 0 (0.0) | 0 (0.0) | 1 (20.0) | 2 (40.0) | 0 (0.0) | 5 (100.0) | 1 (20.0) | 2 (40.0) | 2 (40.0) |
| **LGD (progressors) n=14** | 10 (71.5) | 5 (35.7) | 8 (57.1) | 9 (64.3) | 12 (85.7) | 14 (100.0) | 13 (92.9) | 4 (28.6) | 10 (71.4) |

**Supplementary Table 2.** Logistic regression coefficients and p-values of p53 and aneuploidy for predicting any progression.

|  | **Coefficient** | **Standard Error** | **P-value** |
| --- | --- | --- | --- |
| **Intercept** | 0.23 | 0.048 | 0.000004 |
| **p53 +ve** | 0.19 | 0.089 | 0.037 |
| **Aneuploidy +ve** | 0.39 | 0.126 | 0.002 |


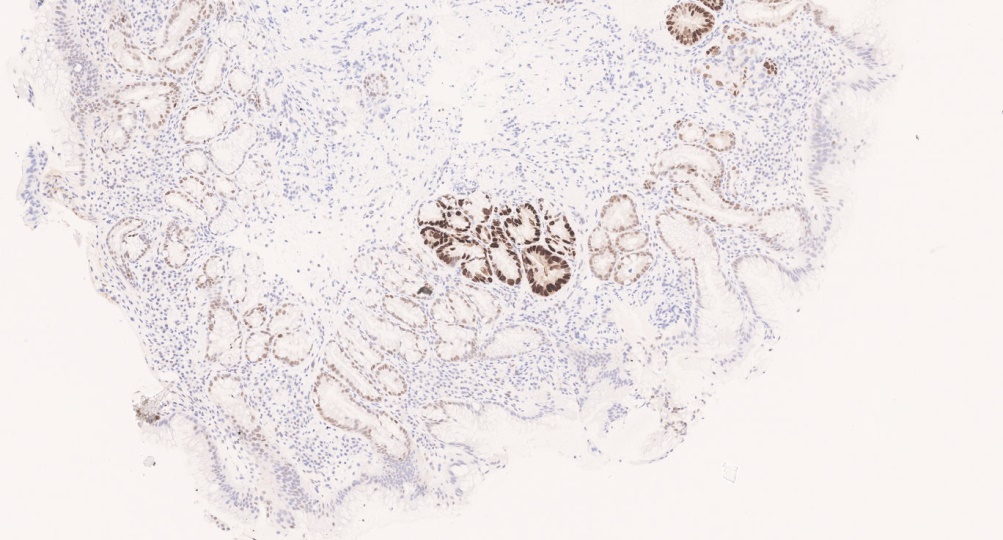

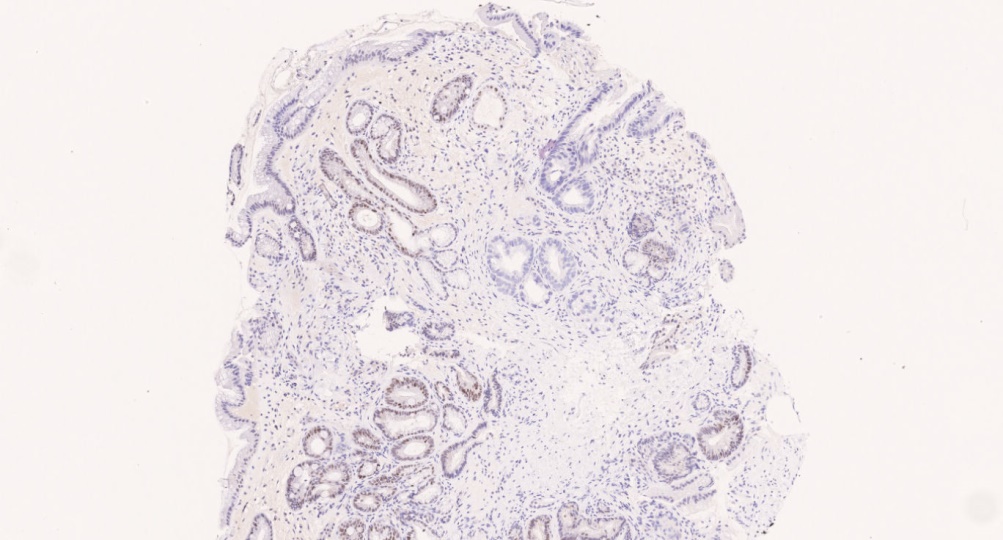

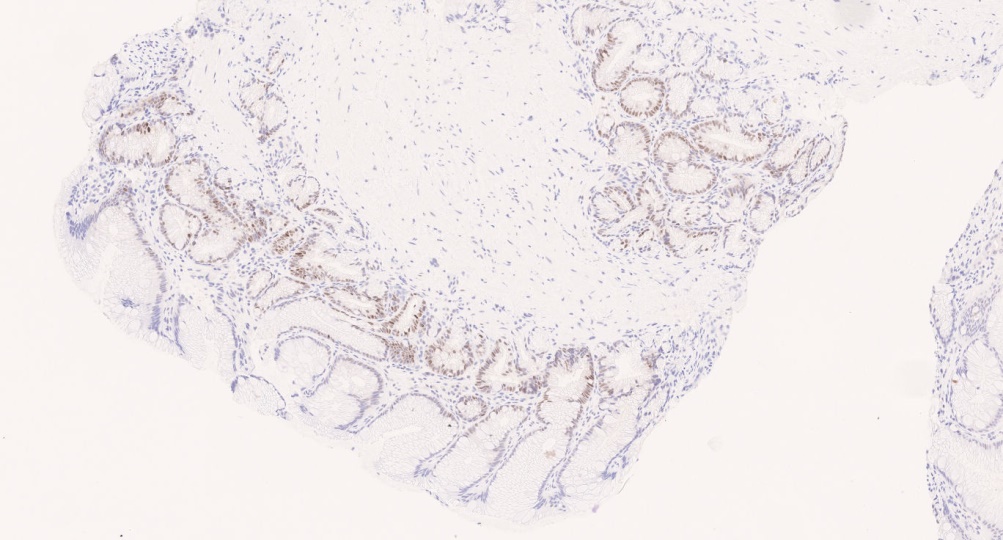


**a**

**b**

**c**

**Supplementary Figure 1. Patterns of p53 staining in Barrett's oesophagus.**

a. Normal pattern of nuclear staining; b. Aberrant pattern with focal protein over-expression (arrow) in a case with basal crypt low-grade dysplasia surrounded by normal background staining; c. Aberrant pattern with focal loss of p53 staining (arrow) surrounded by normal background staining in a case with low-grade dysplasia. These pictures were kindly provided by Dr Maria O’Donovan, Department of Pathology, Cambridge University Hospital.
